# Premenopausal women with breast cancer in the early post-partum period show molecular profiles of invasion and are associated with poor prognosis

**DOI:** 10.1007/s10549-023-06956-6

**Published:** 2023-05-09

**Authors:** Vidya P. Nimbalkar, V. P. Snijesh, Savitha Rajarajan, Annie Alexander, Rohini Kaluve, Rakesh Ramesh, B. S. Srinath, Jyothi S. Prabhu

**Affiliations:** 1 Division of Molecular Medicine, St. John’s Medical College, St. John’s Research Institute, Bangalore, Karnataka India; 2 Centre for Doctoral Studies, Manipal Academy of Higher Education (MAHE), Manipal, Karnataka India; 3 Department of Surgical Oncology, St. John’s Medical College and Hospital, Bangalore, Karnataka India; 4 Department of Surgery, Sri Shankara Cancer Hospital and Research Centre, Bangalore, Karnataka India

**Keywords:** Post-partum breast cancer, Invasion, Prognosis, Time since last childbirth

## Abstract

**Purpose:**

Young premenopausal women develop breast cancer (BC) within 5–10 years of the last childbirth, known as post-partum breast cancers (PPBC), often present with aggressive disease. The exact mechanisms that lead to poor prognosis in these patients are largely unknown.

**Methods:**

We have evaluated the association of clinical and reproductive factors with BC in a cohort of women ≤ 45 years (*N* = 155) with long-term follow-up. Based on duration since last childbirth (LCB), grouped patients into PPBC1 (LCB ≤ 5 years), PPBC2 (LCB between 6 and 10 years), PPBC3 (LCB > 10 years), and NPBC (age-matched nulliparous BC patients). We compared disease-free survival and hazard associated with recurrence/metastasis between the groups. RNA sequencing of tumor samples was performed from three parous groups (*n* = 10), and transcriptomic data were analyzed for differentially expressed genes and altered pathways.

**Results:**

Women in the PPBC1 group had an early menarche and late age at first and last childbirth compared to other groups. Survival analysis within lymph node-positive tumors showed that PPBC1 tumors had a worse prognosis than PPBC2 and NPBC tumors (*p* = 0.015 and *p* = 0.026, respectively). Clustering of the differentially expressed genes between the groups showed distinct expression in early PPBC (E-PPBC) tumors. Pathway analysis revealed upregulation of invasive-related pathways along with T cell exhaustion, extracellular matrix remodeling, angiogenesis, and epithelial-to-mesenchymal transition in E-PPBC tumors.

**Conclusion:**

Early PPBC is a unique subtype with aggressive clinical features and distinct biology. Further research is needed to accurately project the risk of recurrence and optimal treatment strategies in these young patients.

**Supplementary Information:**

The online version contains supplementary material available at 10.1007/s10549-023-06956-6.

## Introduction

Breast cancer (BC) is the most prevalent cancer and the leading cause of cancer-related deaths and morbidity among women worldwide. The proportion of cancer in young premenopausal women with BC is higher in low–middle-income countries probably due to changes in lifestyle and reproductive factors [1, 2]. Delays in pregnancy and older age at childbirth are associated with the early onset of BC. Previous literature shows that BC in younger women is usually associated with aggressive clinical features compared to BC in the older population [3, 4]. Aggressive disease associated with higher morbidity and mortality in the younger population poses a substantial public health problem.

Some of the unique subtypes of BC confined only to young women in the premenopausal age group are pregnancy-associated BC which develops during pregnancy and post-partum breast cancer (PPBC), which extends up to 10 years after the recent childbirth. Studies have shown a transient increase in the BC risk during early post-partum period, compared to age-matched nulliparous BC patients [5, 6]. Previous studies have reported that BC diagnosed in early post-partum duration is more aggressive than diagnosed during pregnancy [7, 8]. PPBC is associated with a threefold increase in metastasis and death related to BC [8, 9], affecting approximately one-third or more of younger women with BC globally. Despite such a huge burden, PPBC is not yet considered a distinct class of BC. Tumor staging and biologic subtyping alone may be insufficient to accurately assess the risk of recurrence and optimal treatment strategies in young premenopausal patients with BC.

Post-partum mammary gland involution is mainly investigated in animal models. Apoptosis-induced cell death and extracellular matrix (ECM) remodeling, like wound healing, are known mechanisms of involution. Rodent model studies have demonstrated that during post-partum involution, the ECM is enriched in collagen, fibronectin fragments, and matrix metalloproteinases (MMPs), contributing to increased invasion and metastatic potential of tumor cells [10-13]. Distinct patterns of immune infiltration in PPBC patients compared to nulliparous BC patients suggest an immunosuppressive microenvironment that might contribute to early disease progression [14]. The exact mechanism that leads to poor prognosis in PPBC in humans is unknown.

In the current study, we assessed the tumor characteristics and risk associated with developing PPBC in women ≤ 45 years of age, grouped them based on post-partum duration compared to nulliparous BC patients. We further evaluated differential biological pathways between early (1–5 years) and late post-partum (> 6 years) within age-matched BC patients.

## Materials and methods

### Patient cohort

The study included a retrospective hospital-based cohort of 155 primary BC patients of age ≤ 45 years who did not receive any prior treatment were recruited for the study from two tertiary cancer care hospitals in Bangalore, India. The diagnosis of BC was confirmed histologically. The maximum follow-up duration was 102 months, with a total loss to follow-up of less than 4% and median follow-up duration of 55 months. The institutional ethical review board approved the study. Informed consent was obtained from all the patients. All the clinical and histopathological information was obtained from the patient’s clinical records. Age at menarche, first childbirth (FCB), and last childbirth (LCB) were collected from medical records or were documented based on specific interrogations with the patients. Patients associated with ambiguous data regarding childbirth were excluded from the study. Information regarding treatment and progression of the disease or disease-related events was obtained during follow-up.

The study cohort was subdivided into four subgroups: PPBC1 (PPBC with LCB ≤ 5 years), PPBC2 (PPBC with LCB between 6 and 10 years), PPBC3 (PPBC with LCB > 10 years), and NPBC (age-matched nulliparous BC patients).

Details of the methods used for RNA sequencing, data processing, functional enrichment analysis, and statistical analysis are mentioned in Additional file 1.

## Results

We accessed women with BC aged ≤ 45 years from a retrospective series of 777 tumors. Among these, 155 (20%) were ≤ 45 years with a mean age ± SD of 38.9 ± 5 years. 48% had tumors size > 3 cm and nearly half (45%) were grade 3. Most tumors were lymph node positive (60%) and had a higher proportion of triple-negative BC (29%).

Categorization based on the time since LCB showed 15% (23/155) were within the first 5 years (PPBC1), 25% (38/155) were between 6 and 10 years (PPBC2), 46% (72/155) had tumors 10 years after most recent childbirth (PPBC3), and 14% (22/155) were NPBC.

### Association of reproductive and clinicopathological features among different groups

Examination of the association of reproductive features between the 3 PPBC groups showed PPBC1 patients had a significantly older age at FCB and LCB compared to PPBC2 and PPBC3 (FCB, *p* = 0.007 and *p* < 0.0001; LCB, *p* = 0.020 and *p* < 0.0001, respectively). We also noticed that PPBC2 patients had significantly higher age at FCB and LCB than PPBC3 patients (FCB, *p* = 0.003; LCB, *p* < 0.001). In addition, within the PPBC1 group, 91% of patients were comparatively younger at menarche (Fisher exact test, *p* = 0.047). No association with the number of live births (calculated as parity) was observed among the parous groups. Results are represented in Table 1. There was no association with the family history of BC.

**Table 1:** Association of parous and nulliparous groups with reproductive features

Characteristics	Subgroups	PPBC1 (1–5 years) (*n* = 23) *n* (%)	PPBC2 (6–10 years) (*n* = 38) *n* (%)	PPBC3 (> 10 years) (*n* = 72) *n* (%)	NPBC (Nulliparous) (*n* = 22) *n* (%)	*p* Value
Age at diagnosis (years)	Mean ± SD	35.5 ± 4.5	36.9 ± 4.2	41.5 ± 2.9	37.3 ± 7.3	** < 0.0001**^**a**^
Reproductive factors						
Age at menarche (years)	> 14	01(4.5)	09(23)	17(22)	05(23)	0.27^b^
	≤ 14	21(91)	29(77)	54(77)	16(73)	
	Missing	01(4.5)	0	01(1)	01(4)	
Age at first childbirth (years)	> 23	18(79)	21(56)	25(35)	NA	** < 0.001**^**b**^
	≤ 23	04(17)	17(44)	47(65)	NA	
	Missing	01(4)	0	0		
Age at last childbirth (years)	> 28	14(82)	18(60)	12(23)	NA	** < 0.0001**^**b**^
	≤ 28	03(18)	12(40)	41(77)	NA	
Parity	> 2	01(4)	08(21)	11(16)	NA	0.156^b^
	≤ 2	22(96)	30(79)	61(84)	NA	

*PPBC* post-partum breast cancer, *NA* Not applicable

^a^Kruskal–Wallis test

^b^χ2 Test

*p* < 0.05, considered statistically significant (represented in bold)

Next, we examined the differences in clinicopathological characteristics such as tumor size, grade, lymph node status, lymphovascular invasion, the density of tumor-infiltrating lymphocytes, estrogen receptor, progesterone receptor, and HER2 status between the parous and nulliparous groups. No significant difference was observed among the groups (Supplementary Table 1, Additional file 1).

### Association of post-partum tumors with prognosis

**Fig. 1:**
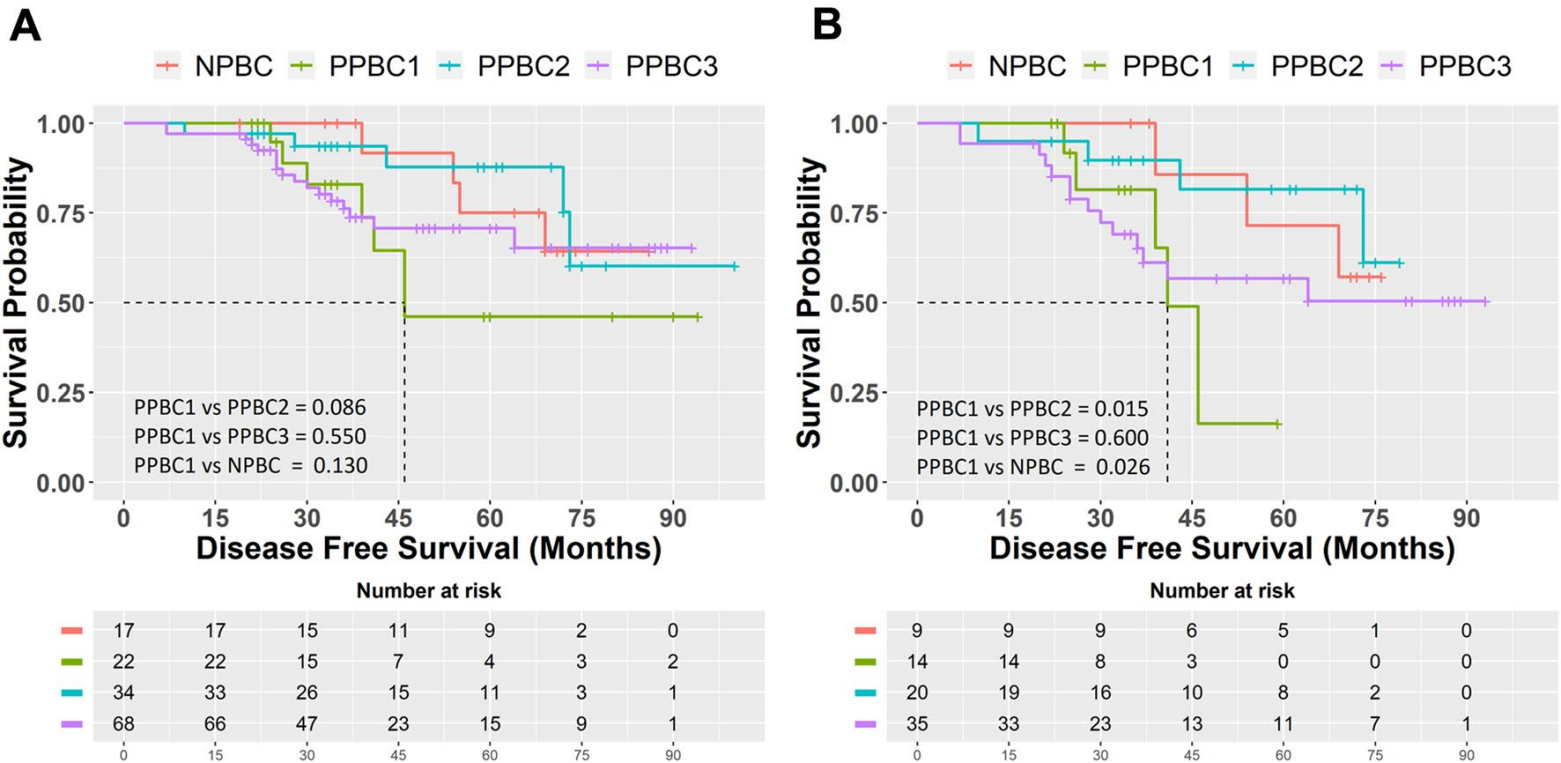
Kaplan–Meier survival analysis for disease-free survival (DFS). **A** Comparison of DFS among four groups in all tumors (*n* = 141). **B** Comparison of DFS among four groups in lymph node (LN)-positive tumors (*n* = 78) showing PPBC1 tumors are associated with poor prognosis compared to PPBC2 and NPBC tumors

Further, we examined the disease-free survival between the parous and nulliparous groups. 141/155 (91%) had complete follow-up information, with the longest and shortest of 102 and 19 months, respectively. Kaplan–Meier survival analysis showed no difference between parous and nulliparous groups (mean survival time ± SD, 72.6 ± 3.3 vs 68.5 ± 4 months, log-rank test, *p* = 0.52). Similarly, no difference in survival probability was observed between the PPBC subgroups and NPBC (mean survival time ± SD for PPBC1, PPBC2, PPBC3, and NPBC was 61.8 ± 8.2, 71.3 ± 3.7, 68.9 ± 4 and, 68.5 ± 4 months, respectively, log-rank, *p* = 0.314) (Fig. 1A).

Next, we performed subset analysis within lymph node-positive tumors alone. 78/141 (56%) were lymph node-positive. Among these, PPBC1 patients (*n* = 14) had a worse prognosis compared to PPBC2 (*n* = 20) and NPBC (*n* = 9) patients (*p* = 0.015 and *p* = 0.026 and mean survival time ± SD for PPBC1, PPBC2, and NPBC was 40.2 ± 2.7, 65.3 ± 5, and 64.3 ± 5.5 months, respectively). Similarly, PPBC3 patients (*n* = 35) had better survival (mean survival time ± SD = 60.3 ± 6 months) compared to PPBC1 patients although not statistically significant (*p* = 0.60) (Fig. 1B).

In concordance with the Kaplan–Meier analysis within lymph node-positive tumors, cox proportional hazard analysis showed that PPBC1 tumors had a higher hazard ratio compared to PPBC2 tumors [HR = 4.83 (95% CI = 1.12–19.61) (*p* = 0.028)] and NPBC tumors [HR = 5.30 (95% CI − 1.006 to 27.92) (*p* = 0.049)] on univariate analysis and [HR = 4.91 (95% CI − 0.68 to 35.13) (*p* = 0.11)] on multivariate analysis.

### Differentially regulated pathways between early and late post-partum groups

**Fig. 2:**
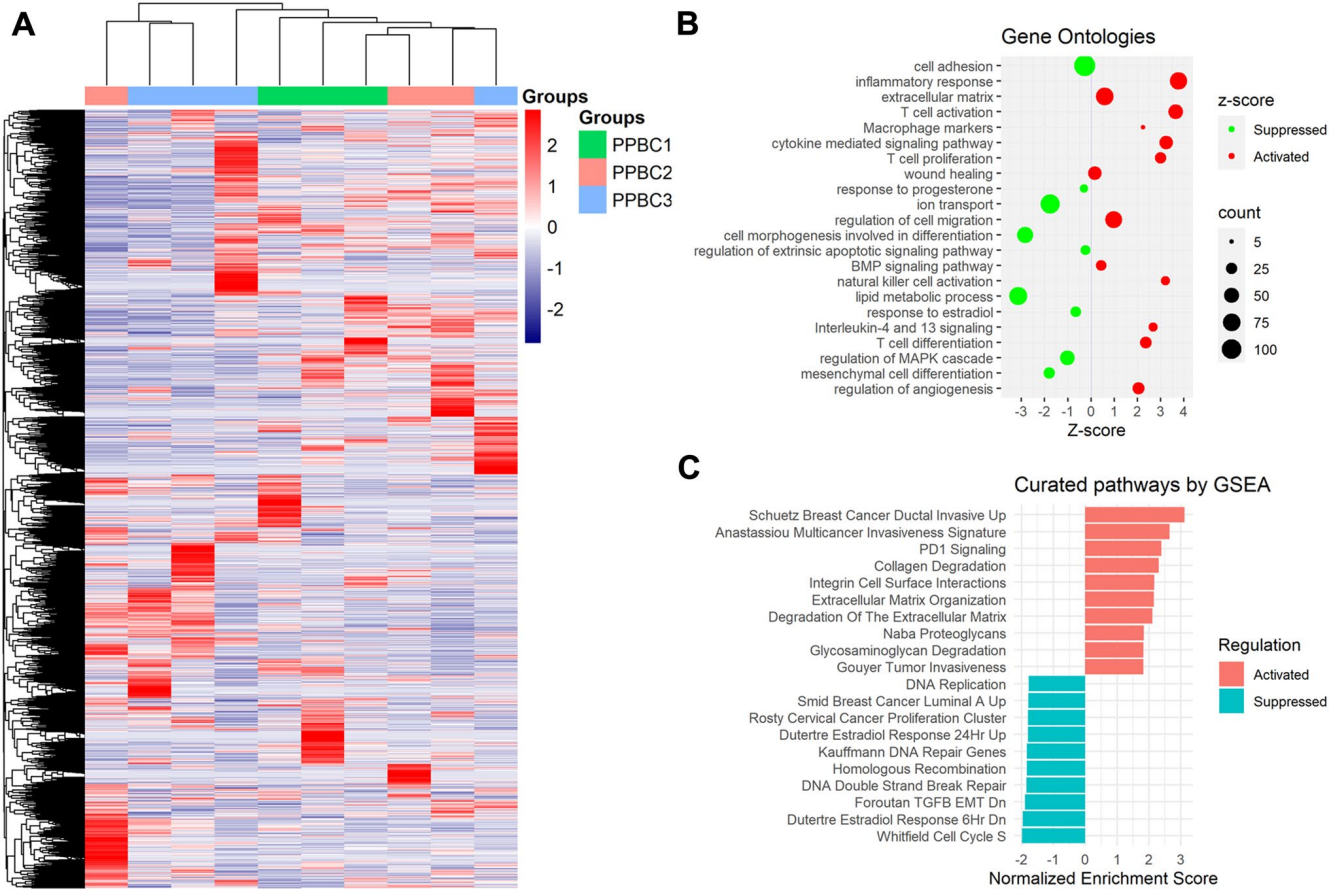
**A** Unsupervised hierarchical clustering of 10 samples across three PPBC groups. **B** Gene Ontologies from enrichment analysis in early PPBC (E-PPBC) tumors (*n* = 3) compared to late PPBC (L-PPBC) tumors (*n* = 7) showing major differentially regulated biological processes. The size of the bubble corresponds to the number of genes involved. C. Curated pathways by gene set enrichment analysis

Further, to understand the biology of tumors in the early and late post-partum period, we carried out RNA sequencing of the PPBC1 (*n* = 3), PPBC2 (*n* = 3) and PPBC3 (*n* = 4) tumor samples. The clinical characteristics of these ten tumors are mentioned in supplementary table 2 (Additional file 1). We obtained 587 DEGs between PPBC1 vs PPBC2 (280 genes upregulated and 307 genes downregulated). Similarly, 894 DEGs were identified between PPBC1 vs PPBC3 (435 genes upregulated, 459 genes downregulated). Unsupervised hierarchical clustering of these ten tumors showed a distinct separation of 8 tumors into three PPBC groups (Fig. 2A). In addition, these samples clustered better based on post-partum duration than ER status or the molecular subtypes (Supplementary Fig. S1, Additional file 1), suggesting that the post-partum period significantly impacts tumor gene expression.

Further, to understand the expression of genes that are concurrently expressed and regulated in the post-partum period since the LCB, we compared the DEGs between PPBC1 vs PPBC2 and PPBC1 vs PPBC3 (Supplementary Fig. S2, Additional file 1). We identified 178 DEGs that were common and overlapping between the groups. Unsupervised hierarchical clustering showed that the expression pattern of these selected DEGs was similar in PPBC2 and PPBC3 (Supplementary Fig. S3A, Additional file 1), and the PPBC1 tumors had a distinct pattern compared to the others. Further, PPBC1 tumors clustered separately in principal component analysis (PCA) (Supplementary Fig. S3B, Additional file 1) from the other two groups (PPBC2 and PPBC3). These results indicated that the post-partum tumors in the immediate vicinity of 5 years, since the LCB were distinctly different in their gene expression from those in the later post-partum period. Hence, we labeled PPBC1 as early PPBC (E-PPBC, post-partum period ≤ 5 years) and PPBC2 and PPBC3 tumors were combined and regrouped as late PPBC tumors (L-PPBC, post-partum duration > 6 years) for further analysis.

Next, we checked the differential pathways regulated between E-PPBC and L-PPBC tumors. There were 745 DEGs between the two groups (287 DEGs were upregulated and 458 DEGs were downregulated). Functional enrichment analysis demonstrated that the E-PPBC tumors were enriched in immune-related processes, ECM organization/degradation, and tumor cell invasion. Gene ontology-based analysis showed that E-PPBC compared to L-PPBC tumors were enriched for immune-related terms, such as leucocyte/lymphocyte/T cell activation, innate and adaptive immune response, and cytokine-mediated signaling, among others. Apart from the immune-related terms, we observed other upregulated processes related to angiogenesis, ECM organization, cell migration, wound healing, and BMP signaling. On the other hand, downregulated processes were cell morphogenesis and differentiation, cell adhesion, ion transport, lipid metabolic processes, response to estrogen and progesterone, regulation of MAPK cascade, and ERBB2-ERBB3 signaling pathway. Figure 2B represents major differentially regulated biological processes in E-PPBC compared to L-PPBC. Curated pathway analysis by GSEA showed that E-PPBC were enriched in tumor invasion along with ECM and its component degradation. In line with the gene ontology analysis, pathways related to cell cycle, DNA repair, and response to steroid hormones were downregulated (Fig. 2C).

To examine if the biological processes observed through gene ontologies were related to the involution process during the normal post-partum period, we obtained gene expression data from normal breast [15] at various time points after childbirth. Based on the time since the LCB, we categorized them into early and late post-partum periods using a similar cut-off of ≤ 5 years. We noticed that most gene ontologies, such as immune-related pathways, cell adhesion, response to steroid hormones, ERBB signaling, cell differentiation and morphogenesis, and angiogenesis were among the commonly enriched ontologies specific to early post-partum duration irrespective of cancerous and non-cancerous conditions (Supplementary Fig. S4, Additional file 1). The major differences observed in the early post-partum period in cancerous conditions were upregulation of angiogenesis, enrichment of macrophage markers, interleukins 4 and 13 signaling, cell migration, and wound healing.

### Estimation of immune cell subtypes between the E-PPBC and L-PPBC groups

**Fig. 3:**
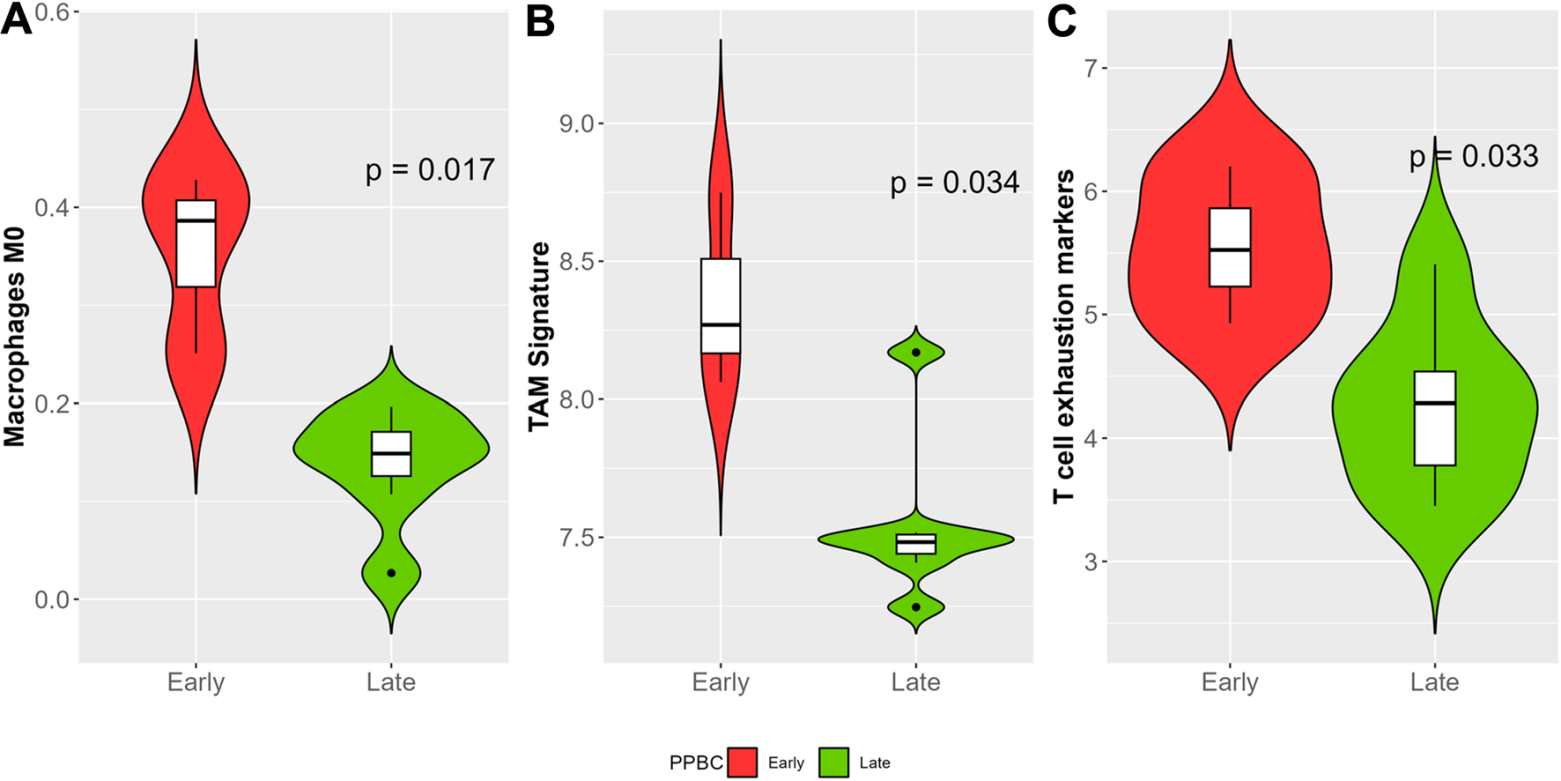
Distribution of immune cell type and immune signatures between early PPBC (E-PPBC) and late PPBC (L-PPBC) groups. **A** shows an increased proportion of M0 macrophages. **B** Increased tumor-associated macrophage (TAM) signature. **C** Increased T cell exhaustion (T cell exhaustion signature included PD-1, PDL1, PDL2, CTLA4, TIGIT, IDO1, IDO2, and TIM3 expression) in E-PPBC

As GSEA analysis showed predominant upregulation of immune-related pathways in E-PPBC tumors compared to L-PPBC, we examined the immune cell subtypes within each group of tumors. We estimated the immune cell infiltration between the groups using deconvolution-based methods using CIBERSORT, xCell, EPIC, MCP-counter, and quanTIseq. Estimating the cell components is important to understand the distinct tumor immune microenvironment. Comparison of the cell types between the two groups showed that the significantly higher cell types (*p* < 0.05) in E-PPBC were M0 (CIBERSORT, xCell, MCP-counter), M1 (xCell, quanTIseq) and M2 (xCell) macrophages, and natural killer cells (xCell, EPIC). The proportion of immature dendritic cells (xCell) was significantly (*p* < 0.05) reduced in E-PPBC tumor samples. The distribution of M0 macrophages in E-PPBC and L-PPBC tumors is represented in Fig. 3A. Further, the proportion of tumor-associated macrophages (TAM) between E-PPBC and L-PPBC tumors was analyzed using 36 gene TAM signatures [16] (Fig. 3B). The distribution of other cell types did not differ between the two groups.

The analysis of differential pathways showed that T cell activation, proliferation, and differentiation were significantly upregulated (Fig. 2B) in E-PPBC tumors. To assess the T cell functionality, we used the T cell exhaustion signature (PD-1, PDL1, PDL2, CTLA4, TIGIT, IDO1, IDO2, and TIM3) [17]. A comparison of the score distribution between the two groups showed E-PPBC tumors had significantly higher T cell exhaustion scores suggesting that these tumors are likely to have pro-tumorigenic microenvironment (Fig. 3C).

### Alterations in key pathways involved in tumor progression in E-PPBC

**Fig. 4:**
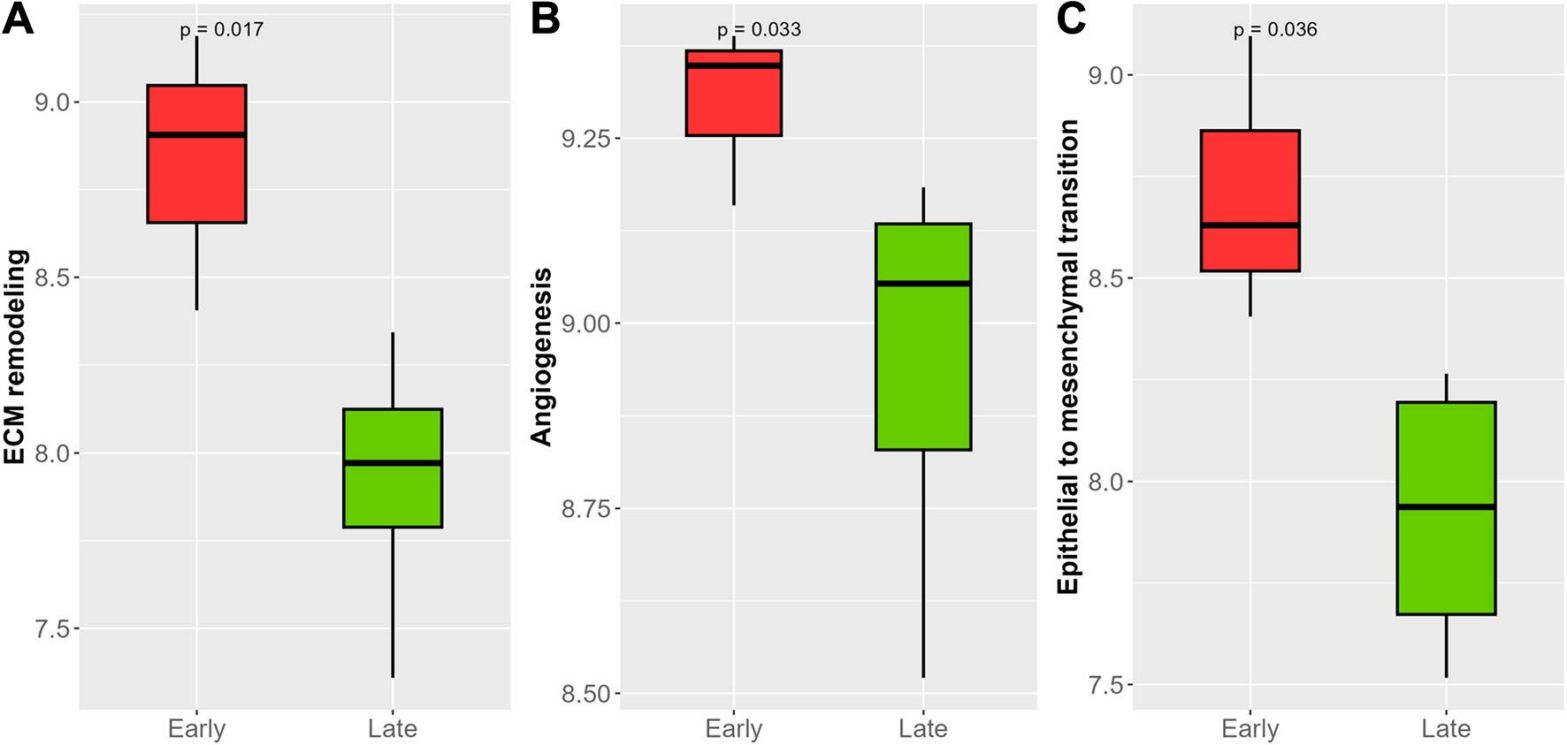
Bar diagrams showing key cancer hallmark pathways upregulated in early PPBC (E-PPBC) tumors compared to late PPBC (L-PPBC) tumors. **A** Extracellular remodeling. **B** Angiogenesis. **C** Epithelial-to-mesenchymal transition

We further examined important cancer hallmark pathways contributing to an aggressive disease, such as ECM remodeling, angiogenesis, epithelial-to-mesenchymal transition (EMT), hypoxia, and stem cell phenotype, using the pathway-specific genes from the molecular signature database. The DEGs mapped with the signature gene sets are mentioned in supplementary Table 3 (Additional file 1). We evaluated ECM remodeling between E-PPBC and L-PPBC groups. DEGs that mapped with selected ECM gene signatures included ECM proteins, ECM regulators, and secreted factors involved in remodeling. E-PPBC tumors were significantly enriched (*p* = 0.017) in the ECM remodeling signature (Fig. 4A).

Tumor growth and metastasis depend on angiogenesis. As mentioned in Fig. 2B, the regulation of angiogenesis was an upregulated process in E-PPBC by GSEA analysis. We further validated this biological process using the angiogenesis gene signature. There was significantly increased (*p* = 0.033) angiogenesis in E-PPBC compared to L-PPBC tumors (Fig. 4B). Additionally, we observed E-PPBC tumors were more mesenchymal, which was indicated by a significantly high EMT score (*p* = 0.036) (Fig. 4C). Further, we also checked expressions of hypoxia-related and stemness gene signatures, and no significant difference was noticed between the two groups.

### Invasive nature of the E-PPBC tumors

On enrichment analysis, we identified genes annotated with the term “breast cancer ductal invasive” as being significantly (*q* = 3 × 10^–40^) upregulated, followed by “multicancer invasiveness signature” (*q* = 6.72 × 10–^12^). The top 10 up- and downregulated pathways enriched using GSEA are represented in Fig. 2C. Enriched terms are given in Additional file 2.

**Fig. 5:**
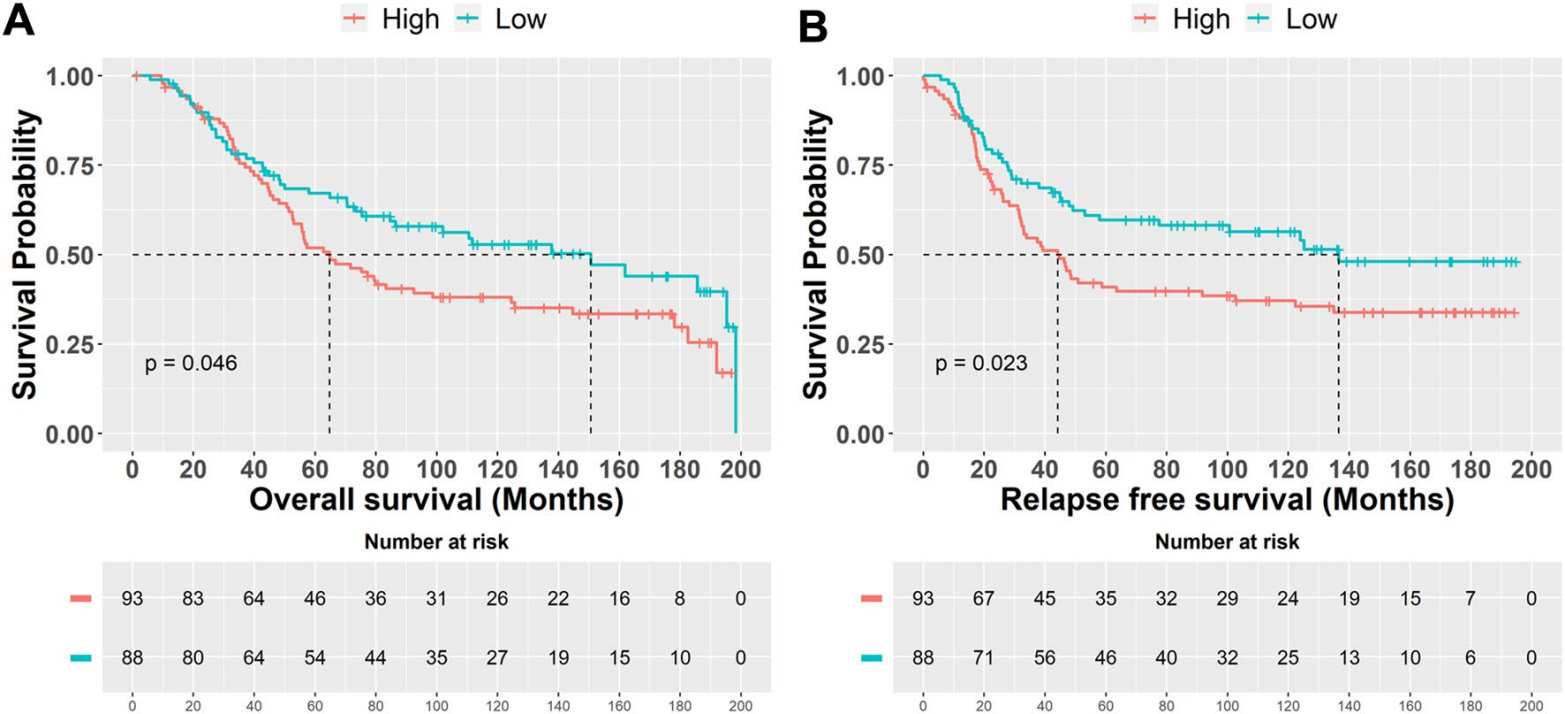
Validation of the invasive signature using the external dataset (METABRIC) by Kaplan–Meier survival analysis in BC patients aged ≤ 45 years. Association of high expression of derived invasive signature with poor outcome. **A** Overall survival. **B** Relapse-free survival

To derive invasion-specific gene signatures in E-PPBC that can predict patient prognosis, 183 leading-edge genes (those genes that “drive” the enrichment score in a GSEA analysis) of “breast cancer ductal invasive” from GSEA analysis were collected. Out of these, 34 upregulated genes overlapped with DEGs of E-PPBC vs L-PPBC. Further, we considered genes falling in the top 25 percentile to derive a smaller subset. Based on this analysis, we derived ten genes, among them nine genes mapped with a public database (METABRIC) (listed in supplementary Table 4, Additional file 1). The gene score was calculated based on the average expression of these nine genes across all samples. We categorized the tumors into high- and low-expression groups using a mean score. Examination of the relapse-free survival in external datasets (METABRIC) in women ≤ 45 years showed tumors with high invasive score had poor survival (mean survival time, high vs low overall survival, 122 vs 99 months, *p* = 0.046 and relapse-free survival, 117 vs 88 months, *p* = 0.023) (Fig. 5). These results further confirmed that invasive signature derived from E-PPBC is associated with poor prognosis.

To check if the gene signature would vary by subtype of breast cancer, we examined the pattern of expression of these genes in tumors (*n* = 10). Further subgrouping was identified by the IHC classification and PAM50 subtypes. Supplementary Fig. S5A (Additional File 1) shows the distribution of the tumors within the subclasses. As seen in the figure, distribution of the genes was clearly different between E-PPBC and L-PPBC and not different between ER + and TNBC tumors. PAM50 subtypes were also distributed in both E-PPBC and L-PPBC tumors without any subtype specificity. We further explored a larger set of tumors, [METABRIC, less than ≤ 45 years (*n* = 248)] and examined the expression of nine chosen genes in these tumors. Tumors were divided into invasive signature high and low (based on median value of mean of 9 genes) due to the lack of information on LCB. Clear pattern emerged between gene signature high and low tumors (Supplementary Fig. S5B, Additional File 1), while no pattern was visible by the ER + /TNBC classes or PAM50 subtypes. These results indicate that invasive signature does not vary by the subtype of the tumors.

## Discussion

Epidemiological studies have demonstrated the dual role of pregnancy on the risk of BC in a time-dependent manner. The risk of BC increases transiently after pregnancy, followed by a period of long-term protection [5, 18]. Involution-associated changes in the mammary gland post-pregnancy induce a pro-tumorigenic environment favorable for tumor development. As these changes are more pronounced during the E-PPBC, we examined the changes in the biological pathways altered in E-PPBC compared to those in L-PPBC. In line with other large cohort studies [19, 20], our findings show an increased risk of metastasis in BC patients within 1–5 years of post-partum duration. Early menarche and older age at first and last childbirth were observed in E-PPBC tumors compared to other groups. No differences were observed for various clinicopathological characteristics among PPBC and NPBC patients in concurrence with previous studies, indicating no selection bias. Earlier reports have shown PPBC tends to have more lymph node-positive disease [19, 21]. Similarly, we also found a poor prognosis confined to lymph node-positive BC patients. Our results confirm post-partum duration as an independent prognostic factor and should be factored into the clinical care of young women with BC.

Gene expression studies done previously in PPBC have compared parous to nulliparous breast tissue in normal as well as cancerous conditions [14, 15, 22] without much emphasis on differentially regulated pathways and biological processes between E-PPBC and L-PPBC. Pereira et al. reported that transient genes upregulated in the normal early post-partum period were mainly immune response genes. In contrast, genes that remained upregulated in the late post-partum period were mainly involved in development/differentiation. We found similar results, showing upregulation of immune-related signaling in E-PPBC and differentiation and morphogenesis-related pathways in L-PPBC. Comparison of gene expression patterns between the normal post-partum period and E-PPBC showed enrichment for similar gene ontology terms indicating these biological processes were specific to early post-partum changes [15]. Other studies have characterized immune cell infiltration in involuting normal breast tissue and reported a transient increase in the immunosuppressed microenvironment [10, 23, 24]. In concordance with these studies, we observed increased expression of T cell exhaustion signatures in E-PPBC tumors indicating a higher proportion of the non-responsive T cell population supportive of the immunosuppressive tumor microenvironment.

We have examined hallmark pathways involved in cancer development and progressions, such as ECM remodeling, angiogenesis, and EMT, and observed they were upregulated in E-PPBC tumors. ECM remodeling is an important mechanism in tumor growth, differentiation, migration, and invasion [25]. ECM components are chemotactic and attract endothelial and immune cells, such as macrophages and neutrophils [26]. EMT and angiogenesis are two other critical processes influencing tumor growth and metastasis. Alteration in these pathways paves the way for tumor cell invasion, which is the first key step in the metastatic cascade of tumors.

Evaluating invasive signatures in BC is mostly confined to identifying ductal carcinoma in situ for its invasive potential [27-29]. Development of clinically relevant human invasive signatures (HIS) using metastasizing tumor cells have been attempted [30-33]. These signatures were enriched in genes that regulate embryonic development, EMT, DNA replication, repair, and resistance to treatment providing mechanistic insights into BC invasion and metastasis [30, 31]. In the current study, we have identified invasive signature in E-PPBC tumors from transcriptomic analysis of whole tumor samples. Analysis of the differentially regulated genes between the E-PPBC and L-PPBC in our study showed ‘breast cancer invasive’ and ‘multicancer invasive’ as the topmost enriched terminologies, followed by ECM organization and degradation, among the other enriched pathways. We then derived an invasive signature from the top quartile of the highly enriched pathway that was differentially regulated between E-PPBC and L-PPBC. These genes were mainly involved in regulating ECM remodeling, cell adhesion, migration, EMT and immune/inflammatory functions. The derived invasive signature’s ability to differentiate overall and relapse-free survival in women ≤ 45 years from public datasets further confirms the enrichment of invasive phenotype in E-PPBC tumors.

Identifying invasive phenotype in E-PPBC may have important biological and therapeutic implications. Firstly, gene expression profile-based tests may not accurately identify risk in these tumors [34-36] as these tests are heavily biased with a predominance of proliferation-associated genes. While we identified invasive pathways upregulated in E-PPBC, our analysis did not find any difference in the expression of proliferation genes between the groups. Earlier studies have shown that the invasive cancer cell population is characterized by downregulation of proliferation and cell cycle genes while upregulation of motility, invasion, and DNA repair genes [30]. Our analysis showed that PAM50-based subtyping of these tumors also partially overlapped with the IHC-based classes, further confirming the inaccuracy of existing methods for identifying molecular heterogeneity of these tumors. Secondly, commonly used cytotoxic chemotherapeutic agents in neoadjuvant settings have previously been shown to induce cell damage and inflammatory wound healing, promoting cell invasion and metastatic dissemination [37]. Given these facts, treating E-PPBC tumors with neoadjuvant chemotherapy can potentially act as an accelerator for the metastatic process contributing to poor prognosis despite being treated with currently accepted therapeutic guidelines. This hypothesis needs to be carefully examined further in clinical trials. Whether these tumors might respond to immunotherapy is speculative at this point. Our analysis showed the higher presence of exhausted T cells in the aggressive E-PPBC tumors suggesting tumorigenic environment. Recent studies indicate the presence of subpopulations of precursor exhausted T cells within the exhausted T cells which respond better to immune check point inhibitors [38, 39]. Further exploration and detailed molecular profiling are necessary to identify tumors enriched for these cellular compositions to derive benefit from immunotherapy for PPBC tumors.

The small numbers limit our study for clinical data analysis and exploration of biological pathways. With the limited number of fresh frozen samples that qualified for in-depth RNA sequencing, we could not perform subtype-specific analysis. The proportion of ER-positive and -negative tumors across three PPBC groups was also variable. However, no correlation was found between IHC subtypes and PPBC groups, indicating our analysis is unlikely to be biased by including both ER-positive and -negative tumors. Additionally, unsupervised hierarchical clustering grouped samples better based on post-partum duration rather than tumor subtype or ER status. The lack of carefully documented information on age at last childbirth in large public datasets prevents identifying PPBC tumors and exploring our findings in other series. Multiple institute-based collaborative efforts are required to conduct specific PPBC cohort studies to validate our findings further.

## Conclusion

Our study reiterates the association of E-PPBC with poor prognosis. Further, it demonstrates that it is characterized by immunomodulation and alterations in several key hallmark pathways favoring tumor cell invasion and leading to early metastasis in these tumors. Understanding the mechanisms of tumor promotion that occur in the post-partum duration will lead to the development of treatment and, possibly, effective prevention strategies for young premenopausal women. We emphasize that the time since recent childbirth should be considered a prognostic biomarker in premenopausal BC.

## Supplementary Information

Below is the link to the electronic supplementary material.

Supplementary file1 (DOCX 1504 KB)

Supplementary file2 (XLSX 30 KB)

## Data Availability

The datasets generated and analysed during the current study are available in the Gene Expression Omnibus with accession number GSE223470 (https://www.ncbi.nlm.nih.gov/geo/query/acc.cgi?acc=GSE223470).

## Abbreviations

BC: Breast cancer
CI: Confidence interval
DEGs: Differentially expressed genes
DFS: Disease-free survival
ECM: Extracellular matrix
EMT: Epithelial-to-mesenchymal transition
E-PPBC: Early PPBC
FCB: First childbirth
GSEA: Gene set enrichment analysis
HR: Hazard ratio
LCB: Last childbirth
L-PPBC: Late PPBC
MMPs: Matrix metalloproteinases
NPBC: Nulliparous breast cancer
PPBC: Post-partum breast cancer
TAM: Tumor-associated macrophages
