# Supplementary material for: Premenopausal women with breast cancer in the early post-partum period show molecular profiles of invasion and are associated with poor prognosis
Source: Breast Cancer Res Treat. 2023 May 9;200(1):139–49. doi: 10.1007/s10549-023-06956-6 (PMC10224850; doi:10.1007/s10549-023-06956-6)
Supplement: Supplementary file 1 — Supplementary file1 (DOCX 1504 KB) [file 10549_2023_6956_MOESM1_ESM.docx]

**Additional data**

**Materials and Methods**

**RNA sequencing and data processing**

RNA sequencing of the PPBC1 (n=3), PPBC2 (n=3) and PPBC3 samples (n=4) samples were performed. Total RNA was extracted from 10 fresh frozen tissue samples stored in liquid nitrogen using Tri reagent (Sigma Aldrich, cat no. T9424). 2-3 sections of 20 µm thickness were used per tumor. Total RNA quality and quantity was assessed using Agilent 4150 TapeStation system (RNA screentape) and Qubit 4 Fluorometer (Qubit DNA BR assay kit). RNA quality was assessed using DV200 values. All ten samples had a DV200 value >27% required for in-depth RNA sequencing.

Total RNA (100 ng) was used for RNA-Seq library preparation, using NebNext Ultra II RNA library prep kit for Illumina (E7770L) according to manufacturer's protocols. The quality and quantity of the library were assessed as described previously. High-quality total RNA-Seq libraries were sequenced (2x150 bp) on NovaSeq 6000 V1.5.

The raw data quality control was performed using FastQC [1]. Adaptor contaminants and low-quality reads with a Phred Score of < 20 were removed using TrimGalore [2]. High-quality reads were aligned to the hg38 reference genome using STAR [3], and mapped reads were counted by FeatureCounts [4]. The genes that had zero counts were removed from the analysis. Further, the differential expression analysis was performed using *DESeq2* [5] based on the Wald test scores, followed by the Benjamini-Hochberg procedure for removing false positives in the data. The genes that had an absolute log fold change (FC) of ≥ 1 and a p-value of <0.05 were screened as differentially expressed genes (DEGs). The heatmap representing DEGs was created using r packages like *pheatmap* [6]. The PAM50 molecular subtyping was performed using r package *genefu* [7].

**Functional enrichment analysis**

Gene ontology annotation was performed using the ToppGene suite [8], and Gene Set Enrichment Analysis (GSEA) [9] was performed using the R package *fgsea* (version 1.20) with pre-ranked genes option to derive the strength and regulation of pathways. The "C2: curated gene sets" (c2.all.v2022.1.Hs.symbols.gmt) from the molecular signature database [9, 10] was used for the analysis. We only considered gene sets significantly enriched with a threshold of normalized enrichment score >1.5 or <−1.5 and false discovery rate, q-value < 0.05.

Immune cell infiltration in the tumor microenvironment was assessed using deconvolution-based methods such as CIBERSORT [11], MCP-counter [12], EPIC [13], xCell [14] and quanTIseq [15].

**Statistical analysis**

Statistical analysis was performed using XLSTAT statistical and data analysis solution (Addinsoft, 2022, New York). Descriptive statistics were used to examine the distribution of the clinical variables. The median value of the distribution was used as a cut-off to categorize various reproductive and clinicopathological factors, and their associations with different PPBC and NPBC groups were assessed using the Chi-square test. Disease-free survival (DFS) was calculated as duration from the date of surgery till the first evidence of metastasis/recurrence. Survival probability was calculated by Kaplan Meier survival analysis and was compared between groups using the log-rank test. The risk factor associated with the progression of the disease was calculated using a univariate Cox proportional hazard model. Results are represented as a hazard ratio (HR) with a 95% confidence interval (CI). P-value <0.05 was considered statistically significant.

**REFERENCES**

1. Andrews S, others (2010) FastQC: a quality control tool for high throughput sequence data

2. Krueger F (2015) Trim Galore. A wrapper tool around Cutadapt and FastQC to consistently apply quality and adapter trimming to FastQ files. Babraham Bioinformatics

3. Dobin A, Davis CA, Schlesinger F, et al (2013) STAR: Ultrafast universal RNA-seq aligner. Bioinformatics 29:15–21. https://doi.org/10.1093/bioinformatics/bts635

4. Liao Y, Smyth GK, Shi W (2014) FeatureCounts: An efficient general purpose program for assigning sequence reads to genomic features. Bioinformatics 30:923–30. https://doi.org/10.1093/bioinformatics/btt656

5. Love MI, Huber W, Anders S (2014) Moderated estimation of fold change and dispersion for RNA-seq data with DESeq2. Genome Biol 15:1–21. https://doi.org/10.1186/s13059-014-0550-8

6. Kolde R, others (2012) Pheatmap: pretty heatmaps. R package version 1:726

7. Gendoo DMA, Ratanasirigulchai N, Schröder MS, et al (2016) Genefu: An R/Bioconductor package for computation of gene expression-based signatures in breast cancer. Bioinformatics 32:1097–9. https://doi.org/10.1093/bioinformatics/btv693

8. Chen J, Bardes EE, Aronow BJ, Jegga AG (2009) ToppGene Suite for gene list enrichment analysis and candidate gene prioritization. Nucleic Acids Res 37:W305-11. https://doi.org/10.1093/nar/gkp427

9. Subramanian A, Tamayo P, Mootha VK, et al (2005) Gene set enrichment analysis: A knowledge-based approach for interpreting genome-wide expression profiles. Proc Natl Acad Sci U S A 102:15545–15550. https://doi.org/10.1073/pnas.0506580102

10. Liberzon A, Subramanian A, Pinchback R, et al (2011) Molecular signatures database (MSigDB) 3.0. Bioinformatics 27:1739–1740. https://doi.org/10.1093/bioinformatics/btr260

11. Newman AM, Liu CL, Green MR, et al (2015) Robust enumeration of cell subsets from tissue expression profiles. Nat Methods 12:453–457. https://doi.org/10.1038/nmeth.3337

12. Becht E, Giraldo NA, Lacroix L, et al (2016) Estimating the population abundance of tissue-infiltrating immune and stromal cell populations using gene expression. Genome Biol 17:1–20. https://doi.org/10.1186/s13059-016-1070-5

13. Racle J, Gfeller D (2020) EPIC: A tool to estimate the proportions of different cell types from bulk gene expression data. Humana Press Inc.

14. Aran D, Hu Z, Butte AJ (2017) xCell: digitally portraying the tissue cellular heterogeneity landscape. Genome Biology 2017 18:1 18:1–14. https://doi.org/10.1186/S13059-017-1349-1

15. Finotello F, Mayer C, Plattner C, et al (2017) quanTIseq: quantifying immune contexture of human tumors. bioRxiv 223180:. https://doi.org/10.1101/223180

**Supplementary table 1. Association of parous and nulliparous groups with clinicopathological characteristics**

| **Clinicopathological factors** | **Subgroups** | **PPBC1**  **(1 to 5 years)(n=23)**n(%) | **PPBC2**  **(6 to 10 years)(n=38)**  n(%) | **PPBC3**  **(>10 years)(n=72)**  n(%) | | **NPBC**  **(Nulliparous) (n=22)**  n(%) | **P value** |
| --- | --- | --- | --- | --- | --- | --- | --- |
| T size (cm) | >3 | 06(26) | 15(40) | | 35(49) | 11(50) | 0.179 |
|  | ≤3 | 14(61) | 21(55) | | 30(42) | 08(36) |  |
|  | Missing | 03(13) | 02(5) | | 07(9) | 03(14) |  |
| Grade | Low | 13(57) | 20(53) | | 31(43) | 12(54) | 0.228 |
|  | High | 07(30) | 15(39) | | 35(49) | 05(23) |  |
|  | Missing | 03(13) | 03(8) | | 06(8) | 05(23) |  |
| Lymph node status | Positive | 15(65) | 24(63) | | 39(54) | 14(64) | 0.61 |
|  | Negative | 08(35) | 14(37) | | 33(46) | 07(32) |  |
|  | Missing | 0 | 0 | | 0 | 01(4) |  |
| Stage | Early | 14(61) | 25(66) | | 43(60) | 12(55) | 0.98 |
|  | Late | 07(30) | 11(29) | | 21(29) | 7(32) |  |
|  | Missing | 02(9) | 02(5) | | 08(11) | 03(13) |  |
| Lymphovascular invasion (LVI) | Yes | 12(52) | 16(42) | | 36(50) | 07(32) | 0.517 |
|  | No | 09(39) | 19(50) | | 29(40) | 11(50) |  |
|  | Missing | 02(9) | 03(8) | | 08(10) | 04(18) |  |
| Tumor infiltrating Lymphocytes (TILs) | High | 09(39) | 22(58) | | 39(54) | 08(36) | 0.127 |
|  | Low | 10(44) | 12(32) | | 14(19) | 08(36) |  |
|  | Missing | 04(17) | 04(10) | | 19(27) | 06(28) |  |
| Estrogen Receptor (ER) status | Positive | 15(65) | 22(58) | | 42(58) | 16(73) | 0.60 |
|  | Negative | 08(35) | 16(42) | | 30(42) | 06(27) |  |
| Progesterone Receptor (PR) status | Positive | 15(65) | 22(58) | | 41(58) | 15(68) | 0.75 |
|  | Negative | 08(35) | 16(42) | | 31(42) | 07(32) |  |
| Human epidermal growth factor receptor 2 (HER2) status | Positive | 04(17) | 09(24) | | 22(30) | 06(27) | 0.63 |
|  | Negative | 17(74) | 24(63) | | 40(56) | 15(68) |  |
|  | Equivocal | 02(9) | 05(13) | | 10(14) | 01(5) |  |

P<0.05, considered as statistically significant (represented in bold); PPBC- postpartum breast cancer

**Supplementary table 2. Clinicopathological features of the 10 samples used for RNA sequencing**

| **PPBC groups** | **PPBC category** | **ER status** | **Grade** | **LN status** | **IHC subtype** | **PAM50 subtype** |
| --- | --- | --- | --- | --- | --- | --- |
| PPBC1 | Early | Negative | II | Positive | TNBC | Normal |
| PPBC1 | Early | Positive | I | Negative | HR+ | LumA |
| PPBC1 | Early | Positive | III | Negative | HR+ | Basal |
| PPBC2 | Late | Negative | I | Negative | HER2 amplified | Normal |
| PPBC2 | Late | Positive | II | Negative | HR+ | LumA |
| PPBC2 | Late | Negative | II | Positive | HER2 Amplified | Her2 |
| PPBC3 | Late | Positive | II | Negative | HR+ | Normal |
| PPBC3 | Late | Positive | III | Positive | HER2 amplified | Basal |
| PPBC3 | Late | Negative | III | Positive | TNBC | Her2 |
| PPBC3 | Late | Positive | II | Negative | HER2 amplified | LumB |

**Supplementary table 3. List of the DEGs mapped with the hallmark pathways from molecular signature database**

| **Signature** | **Genes** |
| --- | --- |
| EMT Signatures | DST, TIMP1, ITGA5, IGFBP3, TNFAIP3, ECM1, IL32, MATN2, RGS4, MMP1, OXTR, MMP3, SCG2, APLP1, SGCG |
| Angiogenesis | CD14, DDIT3, ERBB2, HMGB2, HMOX1, IFITM3, IL18, IL1B, IRF1, LEF1, PDCD4, TAP1, TIMP |
| ECM Remodeling | TIMP1, ADAMTSL3, EGLN3, ADAMDEC1, ADAM19, ADAMTS2, CST3, CTSB, CTSH, HTRA4, HTRA3, F7, MMP1, MMP3, MMP7, SERPINC1, PCSK6 |
| Hypoxia | ALDOC, BCAN, DDIT3, HMOX1, IGFBP3, ILVBL, NCAN, NEDD4L, SIAH2, STC2, TNFAIP3 |
| Stemness | GAL, PON1, CDX2, RBP2, LCAT, SST, ADH4, LPA, CYP19A1 |

**Supplementary table 4. Short listed nine genes used as invasion signature**

| **SYMBOL** | **log2FoldChange** | **P value** |
| --- | --- | --- |
| MMP1 | 3.1247609 | 0.001432168 |
| MMP3 | 2.5384273 | 0.026407387 |
| RGS4 | 2.4842818 | 0.009062749 |
| CCR1 | 1.920523 | 0.000500629 |
| HLA-DQA1 | 1.8653139 | 0.0000016 |
| TMEM158 | 1.6739826 | 0.020415247 |
| IGFBP3 | 1.5845726 | 0.022743236 |
| CCL5 | 1.5465715 | 0.011148845 |
| HLA-DQB1 | 1.5132629 | 0.019533088 |


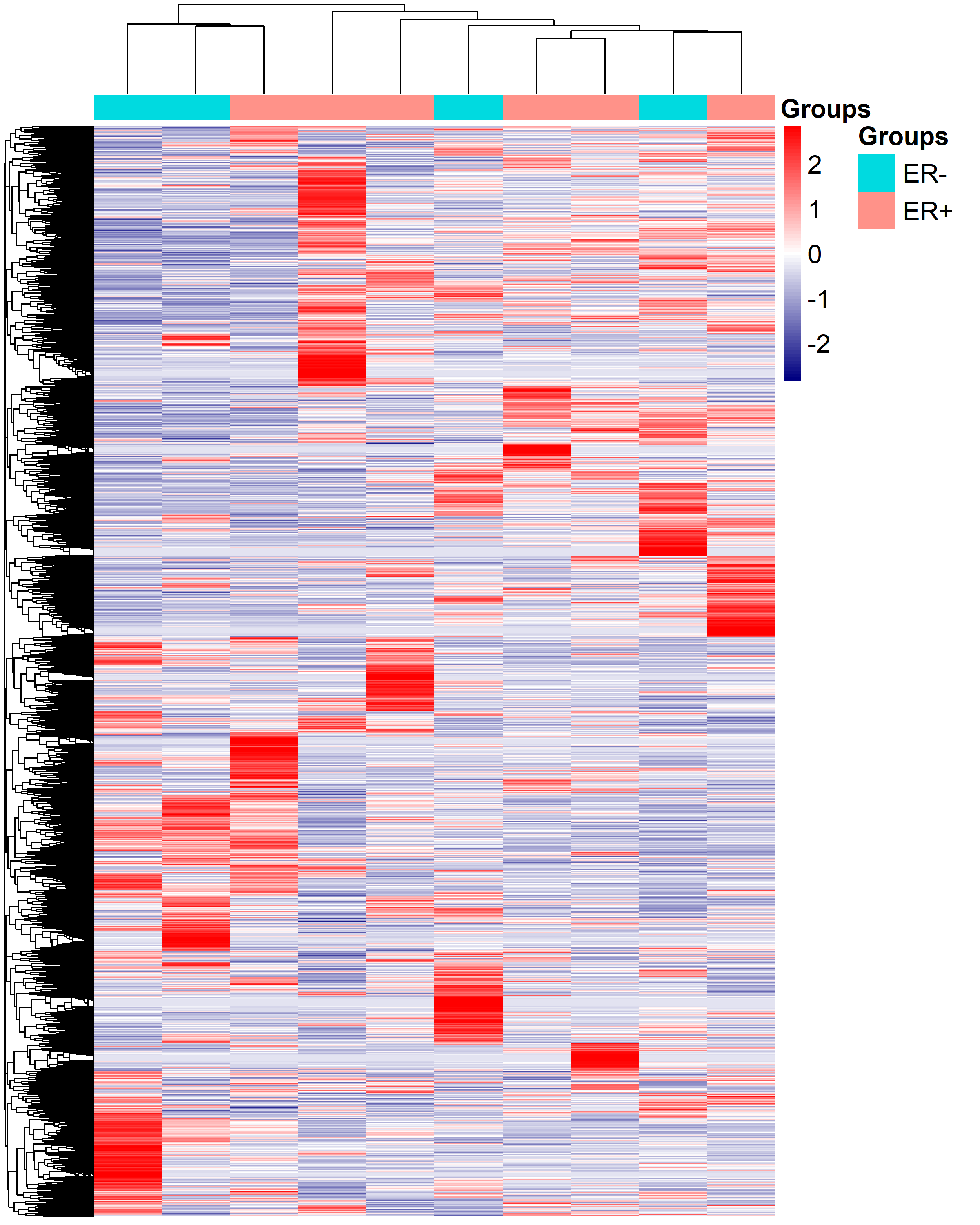


Figure S1. Unsupervised hierarchical clustering of 10 samples across ER positive and negative status.


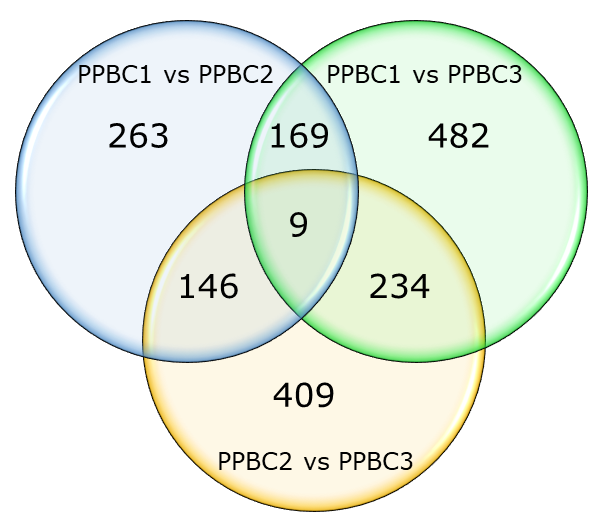


Figure S2. Venn diagram showing unique and common DEGs within PPBC subgroups.


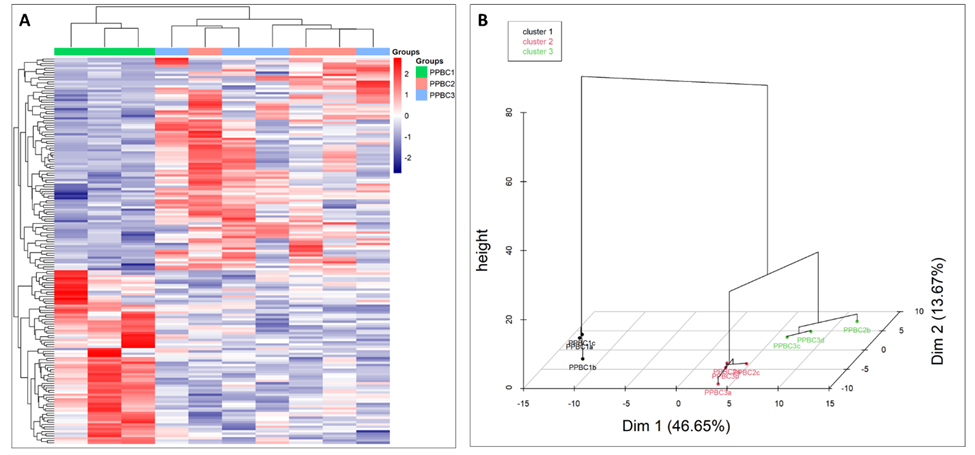


Figure S3. A. Heat map showing unsupervised hierarchical clustering of overlapping differentially expressed genes (n=178) between PPBC1 vs PPBC2 and PPBC1 vs PPBC3 across three parous groups (PPBC1, PPBC2 and PPBC3). B. PCA plot showing clustering of 3 PPBC group sample.


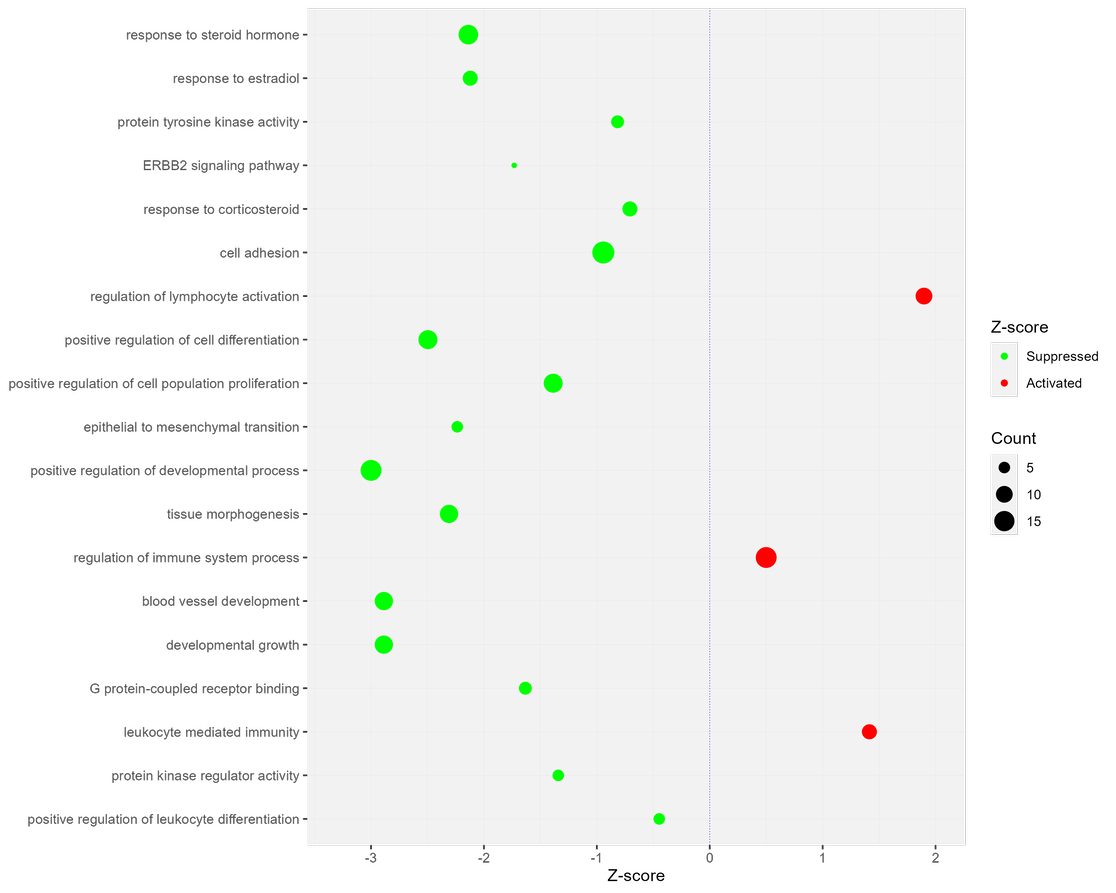
 Figure S4. Gene Ontologies from enrichment analysis in normal early postpartum breast tissue (n=30) compared to late postpartum breast tissue (n=49) showing major differentially regulated biological processes using data from Pereira et al 2019 BCR.


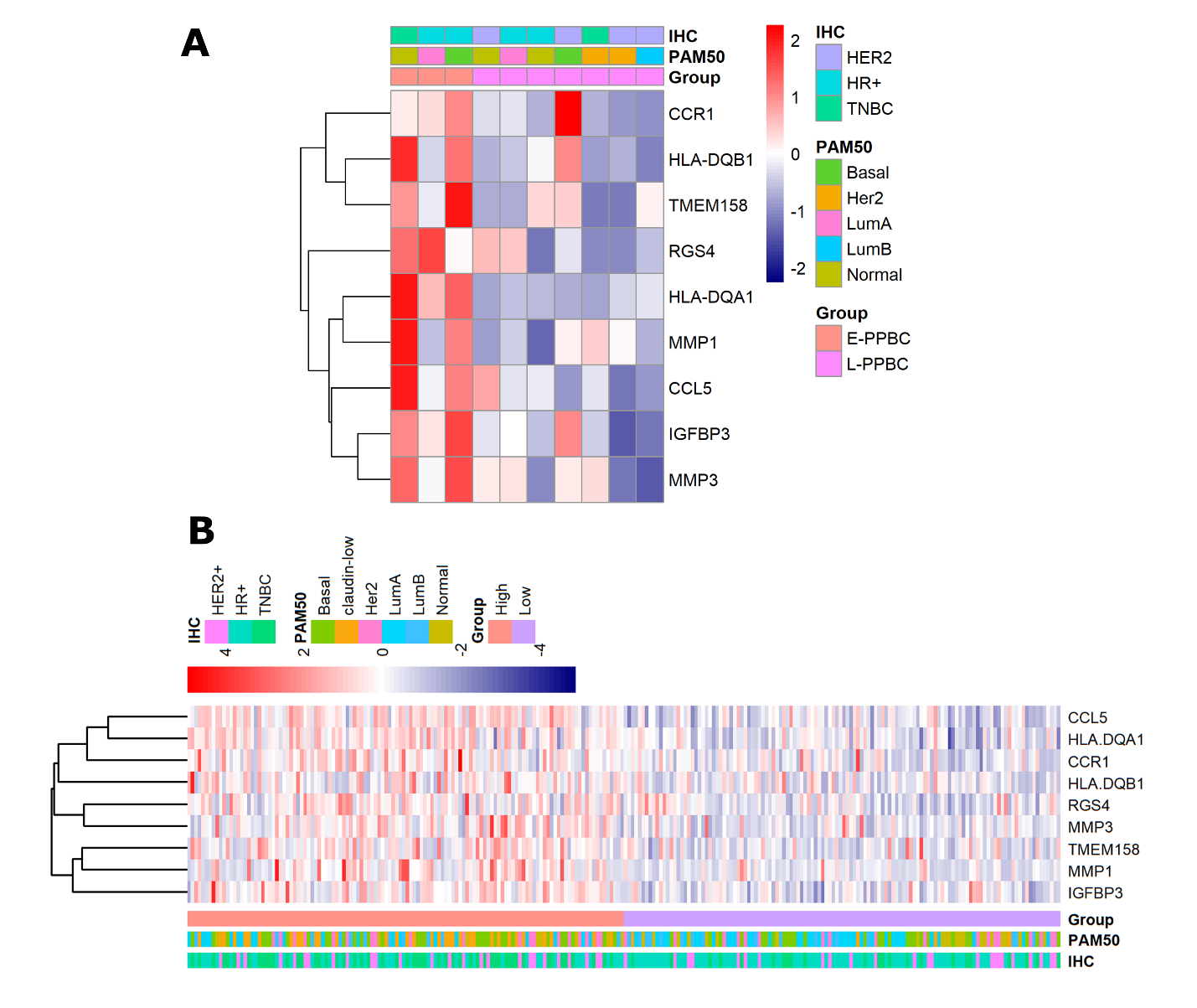


Figure S5. Heatmap representing the expression of invasion specific gene signature across different subtypes (based on molecular subtype and PAM50 subtype) of the breast cancer. A. In study cohort on which RNA sequencing was performed (n=10). B. In the METABRIC cohort with patients aged ≤45 years (n=248)
